# Supplementary material for: The self-reported driving and pedestrian behaviour of adults with developmental coordination disorder
Source: PLoS One. 2024 May 10;19(5):e0301115. doi: 10.1371/journal.pone.0301115 (PMC11086845; doi:10.1371/journal.pone.0301115)
Supplement: Attachment — Submitted filename: Reviewer Responses Plos.docx [file pone.0301115.s001.docx]

**Reviewer #1:** This is an interesting paper which addresses an area of research which is understudied. The manuscript is well written and clear in the methodologies used, however, it is lacking in detail in places and I think a more detailed analysis of the data would really support the conclusions. There are also some other points that I would like the authors to consider before I would be happy to accept this paper for publication:

1. I don't believe the authors have well represented some of the previous studies within this field. For example, the Wilmut and Purcell study cited did consider confidence and also adherence to road regulations (waiting for the green man etc) - what this paper adds that the previous paper did no consider was an inclusion of a control group. These nuanced differences should be really clear so that there is a clear and accurate novelty of the study. This is just one example, a discussion of the relevant papers in more depth would really support the argument for the study.

RESPONSE: We have strived to better represent previous work in this area in the revised submission. We have now added detail from the Wilmut and Purcell (2020) paper reflecting the findings that adults with DCD thought their condition significantly impaired their road crossing ability and reported low self-confidence in road crossing. We have also now stressed the novelty of this study more clearly. Specifically, we highlight this study included a control group and considered other behaviours not covered in the previous work such as the use of mobile phones or headphones while walking in pedestrian settings and the influence of other people on pedestrian behaviours (e.g., following people across the road). Therefore, this paper serves to replicate and supplement the previous work in this area.

2. I would like to know more about both groups. Firstly the group with DCD - what information was given with regards to this, did these individuals have to have a diagnosis of DCD or just suspect that they had DCD? Where any co-occurrences present? This is particularly important as previous work have considered the co-occurrence of ADHD and ASD in road crossing behaviour and so if this study did not consider those co-occurrences some mention of this should be provided in the discussion.

RESPONSE: This is a great point. Participants who reported to have DCD were only eligible to take part if they could confirm they had a formal DCD diagnosis, we have now added the following detail to the ‘Participants section’:-

“One hundred and twenty-eight adults with or without DCD were recruited via an online survey that was distributed by word of mouth, university email addresses, and social media platforms. Participants who reported to have DCD were only eligible to take part if they confirmed they had a formal DCD diagnosis.”

We did not ask about co-cooccurring conditions but have now highlighted this as a limitation and important consideration for further work. Thank you for this suggestion we have added the following text:-

“Finally, a limitation of this work is that we did not account for co-occurring conditions like ADHD that are prevalent in the DCD population and have been previously shown to affect at least some aspects of road crossing behaviour (i.e., looking behaviours; Wilmut & Purcell, 2020). Future research should take greater consideration of co-occurring conditions and their mediating role in pedestrian and driving behaviour.”

3. I would also be interested to know how the two groups were recruited - through the same means? Or were the control group individuals known to the researchers - very little information is given about this.

RESPONSE: Recruitment was facilitated by word of mouth, by sending the survey link to university email addresses and by using social media platforms for both groups. The advert on social media platforms specifically highlighted that we were interested in responses from individuals with and without DCD. We also asked DCD participants recruited via word of mouth to pass the link on to their friends and family. While it’s impossible to give exact numbers related to each recruitment method for each group (there is no way of getting this information from the online survey), we would expect that more individuals with DCD were recruited via social media compared to TD adults. We have added a little more detail on this as stated above.

4. I wonder whether age or years driving influences confidence or behaviour? This is a statistical consideration that the authors have not included - but I would imagine confidence, especially in driving might increase with age in controls - what is the relationship in the DCD group.

RESPONSE: This is an interesting point and we have now run these analyses. For pedestrian behaviour, there was found to be no significant correlation between age and perceived confidence (r = -.057, p =.659) for the DCD group or the for the TD group (r = -.0196, p =.115). For the driving behaviour, there was no significant correlation between age and perceived confidence for the DCD group (r = -.012, p =.938) or for the TD group (r = .048, p =.732). While it may seem intuitive to assume a positive linear relationship between confidence and age in these behaviours, we believe that it’s likely that confidence is more likely to share a curvilinear relationship with age/experience. For example, a novice driver might report low confidence and this confidence may increase with age/experience. However, as people age its likely, in our view, that confidence might diminish during the aging process. These data and SPSS outputs have been uploaded the OSF project file and are freely available.

5. I was really pleased to see that the authors made comparisons between lab based (no risk) and real world situations. However, no discussion is included regarding the limitations of self-report in this context.

RESPONSE: In the final paragraph we previously stated that “There are obvious limitations to self-report measures used in this study also, and the disparity between self-reported and objective measures of driving behaviour and road safety have been documented (Kaye, Lewis, & Freeman, 2018). Therefore, there is a clear need for the examination of the behaviour of individuals with DCD in everyday, real-world environments.” We think that this content is sufficient to address this issue.

**Reviewer #2:** This study investigated the differences between the behaviours of drivers and pedestrians with and without Developmental Coordination Disorder (DCD). While the study subject is very interesting, there are major flaws with the study:

- The objective of the study is not clear. Understanding self-reported behaviour of a group of drivers and pedestrians is not an objective, but a task. What is it that the authors aim to understand (fundamentally) using these self-reported behaviours?

RESPONSE: Thank you for this comment but we never explicitly stated any objective of our study. We instead outlined that ‘The aim of this study was therefore to understand more about the self-reported driving and pedestrian behaviours of adults with DCD.’ Aims refers to the overall goal or purpose of a research study, while objectives are specific statements that describe the steps or actions needed to achieve the aim. Nevertheless, we have now strived to be more concise in outlining the aim of the study. We now state:-

“The aim of this study was therefore to explore the self-reported driving and pedestrian behaviours of adults with DCD to gain an understanding of their perception of the way in which DCD impacts how they navigate these real-world environments”.

- The methodology is not appropriate to address the research gap. As I understand from the manuscript, the identified research gap is that most studies have investigated the behaviours of DCD drivers and pedestrians in simulated environments, which may not be exactly the same as in real-world environments. However and as the authors have mentioned themselves, studying self-reported behaviours has biases and lack of reliability as well. How can the authors claim that studying self-reported behaviours is addressing the gap above?

RESPONSE: We agree that the approach taken here – and indeed the approach taken in studies using simulators – is not perfect but we disagree that this method is not appropriate to answer this research question. There are significant challenges to carrying out this work in real-world driving and pedestrian contexts, which we are sure the reviewer understands. Often researchers will have to make compromises in such designs to shed light on aspects of specific problems, like in these driving and pedestrian scenarios. Self-report and similar methods (e.g., interviews) that aim to capture the lived experience of individuals with DCD are important and should not be undervalued in our opinion. All approaches that provide new insight or replicate previous findings are of value to scientists and those living with DCD. In summary, while we don’t think that our approach can definitively answer this research question (can any?), we do think it adds to the limited research that is currently available on the behaviour of individuals with DCD during driving and pedestrian environments. It is also worth noting that self-report is the most popular method used to gain an understanding of driving and road safety behaviours (Kaye et al., 2018). We have now changed our study aims to hopefully alleviate some of these concerns and to better align our study aims with the approach taken.

Kaye, S. A., Lewis, I., & Freeman, J. (2018). Comparison of self-report and objective measures of driving behavior and road safety: A systematic review. Journal of safety research, 65, 141-151.

- Reliance on self-reported behaviours is even more acute when it comes to the DCD people. As the authors have mentioned, DCD is a neurodevelopmental disorder which, in addition to impaired motor control, may impact cognitive functionality (e.g. spatial awareness, ability to judge distances, reaction time, ability to process multiple stimuli) too. This essentially creates a chicken-egg problem. The self-reported behaviours may well have been influenced by these cognitive disabilities.

RESPONSE: Thanks for this comment. To clarify, individuals with DCD are not classified has having any cognitive/intellectual disabilities – this is a key criterion for their diagnosis. The deficits they experience in executive functions are an integral part of the condition and no doubt contribute to the difficulties they experience in everyday tasks like driving and walking in pedestrian environments. If fact, many people with DCD prefer the term Dyspraxia because they recognise that movement coordination is a small part of the difficulties they experience, particularly in adulthood. We don’t understand the reference to the chicken-egg problem in relation to this because we don’t conclude that the self-reported behaviours are a result of movement impairment, nor do we conclude that they are a consequence of some of the cognitive deficits typical in DCD populations – its likely an interaction between the two.

- What sampling method was used to collect data? From what I can see, the authors have used convenience sampling, which is characterized with insufficient power to identify differences between population subgroups.

RESPONSE: While you are correct in highlighting that a potential limitation of this approach is insufficient power, convenience sampling offers many advantages and is appropriate in circumstances related to the research question outlined here. For example, convenience sampling allows for the collection of preliminary data. This data can be used to estimate effect sizes and variances, which are critical components in determining the required sample size for a more rigorous study with higher statistical power. Second, convenience sampling can be used in pilot studies or initial phases to assess the feasibility of the research questions and methodologies. Insights gained from these preliminary studies can inform decisions about resource allocation for subsequent studies. Finally, and probably most applicable in this context, convenience sampling is often employed when studying niche populations or groups with limited participants. Niche populations may be small or challenging to access (like in DCD), making probability sampling impractical. While the sample size may be limited, the uniqueness of the population can still contribute valuable insights. Understanding the characteristics of these populations might be a critical step before designing larger studies.

This sampling method has been used extensively in DCD related research in driving and pedestrian behaviours as outlined in the introduction section of our manuscript.

- The gender distribution is very different among the DCD and non-DCD samples. How did the authors control for that?

RESPONSE: We are sure that you appreciate it was impossible to control who responded to the online questionnaire. We do see across both groups that females were more likely to respond to the online questionnaire. These sex-based differences in response rates are well documented in the psychological literature (e.g., Becker, 2022). Because of this we do see difference in response rates resulted in females constituting 74% of the DCD sample and 58% of the TD sample – but we don’t think a difference of 16% between groups is problematic. However, we do acknowledge this has a limitation in the dataset. Maybe the biggest difference was in the response rate of male DCD drivers (only 9). While this may be interesting, any sex-based differences in DBQ behaviours was not really the purpose of this research and analysis of sex-based differences in self-reported behaviours would probably be underpowered given these issues.

Becker, R. (2022). Gender and survey participation: an event history analysis of the gender effects of survey participation in a probability-based multi-wave panel study with a sequential mixed-mode design. methods, data, analyses, 16(1), 30.

- The DBQ and PBQ have many items within the same construct. I cannot see the consistency and reliability tests for these items. Did the authors ensure consistency and reliability in the responses?

RESPONSE: We have now added this detail for both questionnaires used in the study. These stats are now included in the data analysis section of the methods and read as follows:-

“Internal consistency and scale reliability was determined for each subscale of each questionnaire using Cronbach's alpha. For the DBQ, errors (α = .76), lapses of attention (α = .80), ordinary violations (α = .84) and aggressive behaviour (α = .81), were found to be reliable and consistent. For the PBQ, scales related to positive behaviour (α = .70), violations (α = .77), lapses of attention (α = .81), and aggressive behaviour (α = .71) were found to be reliable and consistent. These values represent acceptable-to-good internal consistency across all sub-scales (George & Mallery, 2016).”.

George, D., & Mallery, P. (2016). IBM SPSS Statistics Processes for PC. In IBM SPSS Statistics 23 Step by Step (pp. 22-39). Routledge.

- The DBQ and PBQ have many items within the same construct. I can see that the authors have applied the Mann-Whitney tests on the whole construct (e.g. errors, lapses, etc.) but how did they aggregate the questions within he same construct? Did they take the average (which by and of itself may have resulted in lack of statistical power).

RESPONSE: Yes, we took the average score for each construct which is typical when using both surveys in previous research and in the studies validating both questionnaires.
