# Supplementary material for: The self-reported driving and pedestrian behaviour of adults with developmental coordination disorder
Source: PLoS One. 2024 May 10;19(5):e0301115. doi: 10.1371/journal.pone.0301115 (PMC11086845; doi:10.1371/journal.pone.0301115)
Supplement: Attachment — Submitted filename: Reviewer Response R2.docx [file pone.0301115.s002.docx]

Reviewer #2: Thank you for addressing my comments. However, in addition to the response letter, the authors must bring their responses in the manuscript as well:

I would like to see the authors reasoning on the value of their work (i.e. their response to my second comment) in the manuscript as well. I think this is a key discussion that must be in the manuscript to pinpoint the contribution of this work.
RESPONSE: We have now added some of this rationale into the final paragraph of the introduction. We now state:

“The aim of this study was therefore to explore the self-reported driving and pedestrian behaviours of adults with DCD to gain an understanding of their perception of the way in which DCD impacts how they navigate these real-world environments. Due to the significant challenges in carrying out this work in real-world driving and pedestrian contexts, capturing the lived experience of individuals with DCD through self-report will further inform the limited research in this area and help to facilitate future more ecologically valid studies in real-world-contexts.”

Likewise, the authors must bring the discussion around convenience sampling inside the manuscript too.
RESPONSE: We have now added some of the discussion from our response to the manuscript. This is in the participant section of the methods. We state:

“Convenience sampling was used as it offers advantages when studying niche populations or groups that are small or challenging to access, like adults with DCD, which often makes probability sampling impractical. This sampling method has also been used in similar studies on driving and pedestrian behaviour in DCD populations (Kirby et al., 2011; Wilmut & Purcell, 2020).”

The way they have aggregated the questions within the same construct (taking the average) must be stated in the manuscript too.
RESPONSE: We have now added this detail to the ‘Driving behaviour’ and ‘Pedestrian behaviour’ sub-sections within the Measures part of the method section where each questionnaire was explained.
